# Meta-Analysis of Visual Evoked Potential and Parkinson's Disease

**DOI:** 10.1155/2018/3201308

**Published:** 2018-07-11

**Authors:** Song-bin He, Chun-yan Liu, Lin-di Chen, Zhi-nan Ye, Ya-ping Zhang, Wei-guo Tang, Bin-da Wang, Xiang Gao

**Affiliations:** ^1^Department of Neurology, Zhoushan Hospital, Wenzhou Medical University, Zhoushan 316021, China; ^2^Department of Critical Care Medicine, Huzhou Central Hospital, Huzhou 313000, China; ^3^Department of Neurology, Taizhou Municipal Hospital, Taizhou 318000, China; ^4^Department of Nutritional Sciences, The Pennsylvania State University, USA

## Abstract

**Background:**

Previous studies suggested that visual evoked potential (VEP) was impaired in patients with Parkinson's disease (PD), but the results were inconsistent.

**Methods:**

We conducted a systematic review and meta-analysis to explore whether the VEP was significantly different between PD patients and healthy controls. Case-control studies of PD were selected through an electronic search of the databases PubMed, Embase, and the Cochrane Central Register of Controlled Trials. We calculated the pooled weighted mean differences (WMDs) and 95% confidence intervals (CIs) between individuals with PD and controls using the random-effects model.

**Results:**

Twenty case-control studies which met our inclusion criteria were included in the final meta-analysis. We found that the P100 latency in PD was significantly higher compared with healthy controls (pooled WMD = 6.04, 95% CI: 2.73 to 9.35, *P*=0.0003, *n*=20). However, the difference in the mean amplitude of P100 was not significant between the two groups (pooled WMD = 0.64, 95% CI: −0.06 to 1.33, *P*=0.07) based on 10 studies with the P100 amplitude values available.

**Conclusions:**

The higher P100 latency of VEP was observed in PD patients, relative to healthy controls. Our findings suggest that electrophysiological changes and functional defect in the visual pathway of PD patients are important to our understanding of the pathophysiology of visual involvement in PD.

## 1. Background

Parkinson's disease (PD) is one of the most common neurodegenerative disorders in the world. The prevalence of PD is expected to rise steadily in the future as the human population ages [1]. Visual dysfunction is a common nonmotor symptom in individuals with PD, including abnormal contrast sensitivity, motion perception abnormalities, and impaired visual acuity and color vision [2, 3]. Visual dysfunction that occurs in PD is subtle and could be easily demonstrated through electrophysiologic testing, such as the visual evoked potential (VEP). VEP is a potential change recorded in the visual cortex after retinal received light stimulation, which reflects the functional status of the entire visual pathway. VEP latency is less likely to be affected by dopaminergic drugs and seems to be a more sensitive measure of foveal electrical activity than VEP amplitude. It is thought that abnormal latency is due to delayed conduction in visual pathways affected by the process of demyelination and/or plaque formation [2]. The P100 latency of VEP is usually used to determine the abnormalities of the visual pathway due to the relatively small individual difference.

As the pathological hallmark of PD is progressive loss of dopaminergic neurons in the substantia nigra, the human retina also contains dopaminergic amacrine and interplexiform cells, which play a regulatory role. Several observations support the concept that dopamine has a specific function in the retina of primates [4, 5]. The chemical protoxin MPTP (1-methyl-4-phenyl-1-2-3-6-tetrahydropyridine), which produces a clinical parkinsonian syndrome, significantly lowers retinal dopamine. Similarly, intravitreal injection of the neurotoxin 6-hydroxydopamine into aphakic monkeys resulted in abnormalities in both the phase and amplitude of the pattern VEP [6].

Visual dysfunction was observed in some early PD patients not yet undergoing L-dopa therapy, indicating that visual deficiency could be one of the prodromal symptoms of PD [3, 7, 8]. However, previous studies regarding visual dysfunction, as assessed by the VEP, in PD patients versus general populations generated mixed results [8–10], which could be due to small sample sizes of these individual studies (PD case numbers < 50 for all studies). We thus performed a systematic review and meta-analysis to comprehensively assess whether the pattern reversal VEP latency, as the primary exposure, was different between PD patients and controls. However, we also examined other visual indices, including pattern reversal VEP amplitude, intraocular difference of P100 latency, and N75 latency.

## 2. Materials and Methods

This meta-analysis was performed according to the Preferred Reporting Items for Systematic Reviews and Meta-Analyses (PRISMA) statement (PRISMA Checklist in supplementary materials (available here)) [11].

### 2.1. Search Strategy, Study Inclusion Criteria, and Data Extraction

Two of the coauthors (Chun-yan Liu and Ya-ping Zhang) independently searched the literature and extracted the relevant information from the eligible studies. A systematic review of the literature was conducted using the databases PubMed, Embase, and the Cochrane Central Register of Controlled Trials from 1 January 1978 up to 10 May 2016 to identify the relevant studies. We searched the medical subject heading (MeSH) terms “Parkinson's disease” and “visual evoked potentials” in PubMed, respectively, and found out their entry terms. We only included English papers. Retrieved studies were imported into Mendeley Desktop (version 1.16; PDFTron^TM^ Systems Inc.), where duplicate articles were deleted. Titles and abstracts of the remaining studies were independently scanned by Chun-yan Liu and Ya-ping Zhang. The full texts of the potentially relevant reports were then read to determine whether they met the inclusion criteria. The reference lists from all included studies were also examined.

Studies fulfilling the following inclusion criteria were included in the present meta-analysis: (1) participants were adult; (2) study design was a case-control study including a group of diagnosed idiopathic PD; (3) all participants underwent pattern reversal VEP examination, and both visual acuity test and ophthalmologic evaluation showed normal results; (4) information on peak latency of the P100 component was provided; and (5) sample size was greater than 10 in each group. Exclusion criteria were as follows: (1) participants were PD patients with dementia or undergoing deep brain stimulation; (2) authors did not make pattern reversal VEP examination; (3) studies without healthy control group; (4) studies did not provide the P100 data of pattern reversal VEP; (5) review papers; (6) reports published only in the abstract form; and (7) papers were not written in English.

A standardized data extraction form was used to collect relevant information including the name of the first author, publication year, study country, PD diagnosis criteria, mean disease duration, scales used for evaluating motor and cognitive function, number of eyes, mean age, sex, and latency of P100.

Two reviewers (Chun-yan Liu and Ya-ping Zhang) separately evaluated studies, and discrepancies were resolved by discussion. If disagreements existed, in very few cases, two reviewers consulted a third party (e.g., Xiang Gao) until both reviewers reached an agreement.

### 2.2. Study Quality Assessment

We assessed the methodologic quality of included studies based on the Newcastle-Ottawa Scale (NOS) [12] for quality of the case-control study. The NOS uses a star rating system to judge the quality based on 3 dimensions of the study: selection, comparability, and exposure. A study could be scored a maximum of one star for each item numbered within the categories of selection and exposure, while at most two stars could be allocated to comparability. A higher score represented better quality of the study methodology. The maximum score was 4 for selection dimension, 2 for comparability, and 3 for outcome/exposure. A study with a score equal to or higher than 6 was considered of high quality.

### 2.3. Statistical Analysis

Statistical analysis was performed using RevMan (version 5.3; Cochrane Collaboration, Oxford, United Kingdom) and STATA statistical software (version 12.0; StataCorp, College Station, TX, USA). Data that could not be obtained were to be calculated when necessary. For example, when standard deviation (SD) was not available, it was calculated using the sample sizes and standard error. For continuous outcome, means and standard deviations were used to calculate the pooled weighted mean differences (WMDs) and their 95% confidence intervals (CIs). The chi-square test, tau^2^, and the Higgins *I* ^2^ test were used to assess heterogeneity [12]. The *I* ^2^ test is a method for quantifying inconsistency across studies and describes the percentage of variability in effect estimates due to heterogeneity. A value greater than 50% was considered substantial heterogeneity. A fixed-effects model was adopted for the analysis if the *P* value was >0.1 and the *I* ^2^ index was <50%, as these results would indicate no between-study heterogeneity. Otherwise, a random-effects model was applied [13].

Potential publication bias was examined using funnel plots [14]. To evaluate the influence of each individual study on the stability of the overall pooled estimate, we conducted sensitivity analyses by removing each study and observing whether the result changed. Metaregression analysis and subgroup analyses were conducted according to the sample size, publication year, age, sex ratio, and mean PD duration of the cases to explore the potential inherent heterogeneity across the included studies.

## 3. Results

**Figure 1:**
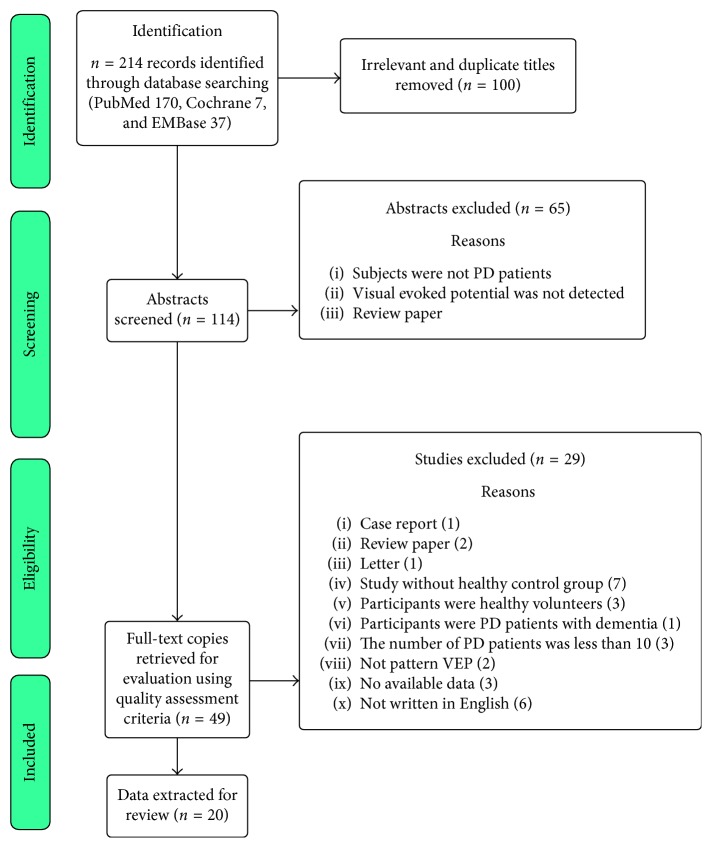
Search and study selection process.

A total of 214 articles were initially identified, and 20 case-control studies which met our inclusion criteria were included in the final meta-analysis (Figure 1; Table 1) [2, 3, 7–10, 15–28]. Seven out of the 20 studies had the NOS score equal to or more than 6, and the mean score was 6.3.

**Figure 2:**
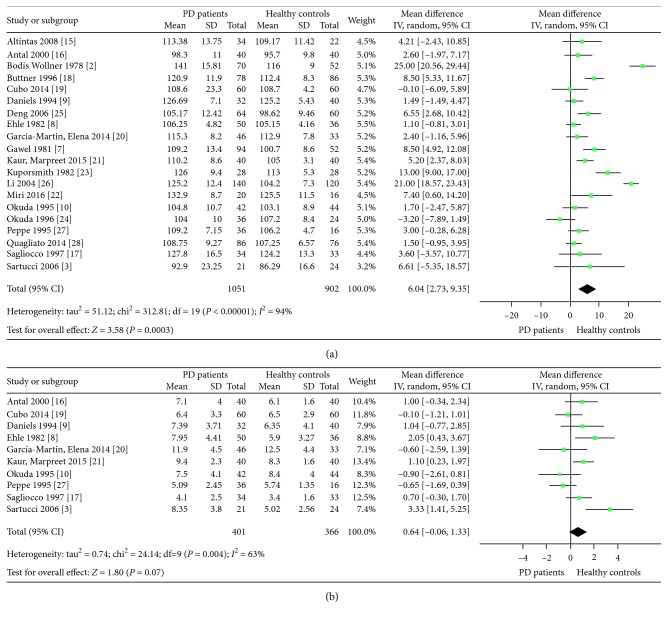
Pooled weighted mean differences in visual evoked potential: (a) P100 latency and (b) P100 amplitude.

Because of significant heterogeneity (*I* ^2^ = 93%) across the included studies, we used the random-effects model to calculate the WMDs. The P100 latency (WMD = 6.04, 95 % CI: 2.73 to 9.35, *P*=0.0003, *n*=20), but not amplitude of P100, was significantly higher in PD patients compared with healthy controls (Figures 2(a) and 2(b)).

Metaregression analysis and subgroup analyses revealed that different sample sizes, sex ratios, and disease durations across studies (all *P*<0.05), but not publication year and age (both *P*>0.05), were possible sources of heterogeneity observed in the current meta-analysis (Table 2). The difference in P100 latency between PD patients and controls was more pronounced in the studies with a large proportion of men and long PD duration patients (*P*-difference < 0.05) than the studies with a small proportion of men and short PD duration patients (*P*-difference > 0.05) (Table 2).

**Figure 3:**
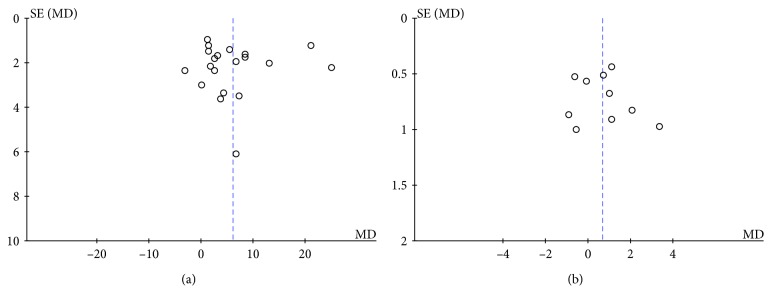
Funnel plots for evaluating the publication bias for visual evoked potential: (a) P100 latency and (b) P100 amplitude.

Sensitivity analyses showed no significant changes in the pooled WMD or 95% CI upon the exclusion of any study, indicating that the overall pooled estimates were stable. The funnel plot did not support existence of publication bias (Figure 3).

## 4. Discussion

In this meta-analysis based on 20 case-control studies, we observed that there is a significant delay in the VEP, as assessed by P100 latency, in individuals with PD, relative to controls. PD is a disorder of the motor system in which there is no obvious clinical involvement of the visual system and with no pathological lesions (e.g., demyelination), that are considered major determinants of the delays in conduction in visual pathways [2]. Our findings could lead to a better understanding of the physiology of human and primate vision: how and where dopamine acts in the elementary retinal processing of signals related to spatial contrast.

The changes in VEP reflect functional damage in the visual pathway of PD patients. Someone also found structural damage in the retina by using optical coherence tomography which revealed thinning of the retinal nerve fiber layer and macula [29] in PD. The deficit is most evident in the annular zone surrounding the fovea, where anatomical studies [30] demonstrated the highest concentration of dopaminergic amacrine cells. Dopaminergic deficiency is related to a VEP delay. The postmortem study of PD's retina observed a decrease in retinal dopamine concentration [31]. The thinned inner retina [32] suggests involvement of the ganglion cell layer and dopaminergic neurons and the inner plexiform layer in PD. Importantly, previous studies suggested that the structural changes correlated significantly with functional changes [15, 19, 21, 22].

We found a significant delay in the average P100 latency in PD patients compared with the healthy controls, and this difference could be modified by different sample sizes, PD durations, and sex ratios across studies. Disease duration and sex difference may be associated with electrophysiologic changes in the visual pathway of patients with PD. Previous studies reported that the functional changes correlated significantly with disease duration and severity of PD [21, 22]. Furthermore, a prolonged mean VEP latency in PD patients was shown to be dependent on the sex of the participants (more evident in men than women) [33]. The difference in the thickness of the skull and the brain volume between men and women could also explain, at least in part, the gender difference in VEP for PD patients.

The mechanism of visual defect in PD may act as follows: pathological changes in PD are not only found in the nigra-striatum system but also in the caudal nuclei, putamen nuclei, hypothalamus, and pontine nucleus ceruleus [30]. This could result in cholinergic system dysfunction that diffusely affects the brainstem auditory pathway. The injuries to these regions cause dopaminergic neuron degeneration and decrease in dopamine production and secretion, which may affect the function of the interplexiform cells and horizontal cells in the retina to undermine the transmission of the visual signals, contributing to the abnormal changes of VEP [18]. Then, dopamine exists in the lateral geniculate body and visual cortex, and the visual cortex also contains acetylcholine and its receptors [34]. Long-term dopamine deficiency could trigger a compensatory decrement of acetylcholine and lead to changes in the VEP latency. A retinal dopaminergic deficiency could also underlie some visual changes in PD. However, the relationship between the decrement of dopamine in the nigra-striatum of PD patients and dopamine dysfunction in the visual pathway needs further study. Alpha-synuclein (*α*-syn) is also expressed widely in the vertebrate retina including humans [35], and it is likely that loss or dysfunction of *α*-syn at this site is responsible for visual symptoms [36].

It is worth noting that VEP measures the integrity of the entire visual pathway. The changes in these potentials in PD may reflect the widespread nature of the biochemical disorder affecting both the retina and central nervous system. Thus, the VEP is a relatively poor method for assessing the anterior visual pathway dysfunction because it is incapable of differentiating impaired macular function from impaired ganglion cell function. Although all the patients were normal on ophthalmological examination, we cannot exclude involvement of the direct visual pathway in PD as a cause of delayed VEP. The pattern electroretinogram (PERG) measures the electrical activity generated by neural and nonneuronal cells in the retina in response to a light stimulus and reflects functional changes in the retina. The PERG would differentiate whether the retinal abnormalities were the cause of the VEP changes. As we expected, some studies reported impaired PERG in PD patients and confirmed the retinal functional defect [20, 21]. However, they still cannot exclude the disorder in the upper visual pathway. Furthermore, both PERG and VEP improve with therapy, but there is an apparent difference: levodopa therapy improves PERG abnormalities to a higher degree than it does VEP deficits [27, 37, 38]. One possible interpretation is that VEP changes in PD represent secondary nondopaminergic, and therefore more chronic, alterations in visual processing. It seems that additional pathology beyond the retina affects visual responses, including VEPs. Although the role of retinal dysfunction seems certain, the contribution of cortical and lateral geniculate impairment to these visual symptoms remains unknown.

This meta-analysis has some limitations, which are as follows: (1) We did not include unpublished studies and those which were not written in English. The current study thus may lose some high-quality evidences. (2) Because most studies did not provide data regarding PD severity, we failed to explore the potential impacts of disease severity on the VEP-PD relation. (3) We cannot address the potential impact of dopaminergic drugs because most PD cases were treated. Previous studies suggested that these treatments could improve contrast sensitivity and reverse VEP delays in PD patients [2, 39, 40]. Failure to control the use of dopaminergic drugs thus would underestimate the true difference in VEP latency between PD patients and controls. (4) Some participants with other neurodegenerative diseases were probably included in control groups, as screenings are not fully stringent so as to exclude early dementias.

## 5. Conclusion

This meta-analysis showed that the P100 latency was significantly longer in PD patients than healthy controls. The application of VEP provides an approach for a more comprehensive evaluation of the visual pathway and a better understanding of the pathophysiology of visual involvement in PD. However, further researches with longitudinal study design and incorporating the pattern electroretinogram assessment are warranted.

## Figures and Tables

**Table 1:** Characteristics and quality of studies included in the meta-analysis.

Author(s) year	Country	Diagnostic criteria	Mean duration (months)	Motor and cognitive function	Number of eyes		Mean age (years)		Sex (men/women)		Newcastle-Ottawa Scale (NOS)			
					PD	HC	PD	HC	PD	HC	Selection	Comparability	Exposure	Total score
Altintaş 2008 [15]	Turkey	UKBB	54.96	UPDRS	34	22	59.29	58.09	9/8	6/5	3	1	3	7
Antal 2000 [16]	Austria	NR	58.1	UPDRS, H&Y, ADL, MMSE	40	40	65.3	59.8	13/7	13/7	2	2	3	7
Bodis-Wollner 1978 [2]	USA	NR	97.8	NR	70	52	62	60	NR	NR	2	1	2	5
Buttner 1996 [18]	Germany	NR	64.8	UPDRS, H&Y	78	86	64.0	62.8	20/19	18/25	2	1	3	6
Cubo 2014 [19]	Spain	UKBB	98.4	SPES/SCOPA, H&Y, MMSE	60	60	65.4	62.9	18/12	15/15	3	1	3	7
Daniels 1994 [9]	UK	NR	NR	NR	32	40	64.36	66.95	NR	NR	2	1	3	6
Deng 2006 [25]	China	CMA	NR	NR	64	60	64	64	20/12	19/11	2	1	2	5
Ehle 1982 [8]	USA	NR	65	H&Y	50	36	64	56	NR	NR	2	1	3	6
Garcia-Martin, Elena 2014 [20]	Spain	UKBB	92.4	H&Y, SE-ADL	46	33	70.72	69.67	29/17	21/12	3	2	3	8
Gawel 1981 [7]	England	NR	NR	NR	94	52	67	63	30/17	11/15	2	1	3	6
Kaur, Marpreet 2015 [21]	India	UKBB	69.6	H&Y, UPDRS	40	40	58.6	58.4	NR	NR	3	1	3	7
Kuporsmith 1982 [23]	USA	NR	NR	H&Y	28	28	62	58	NR	NR	2	1	2	5
Li 2004 [26]	China	CNCESD	56.4	UPDRS, H&Y, HDS	140	120	62.2	59.9	32/38	28/32	3	1	3	7
Miri 2016 [22]	USA	UKBB	NR	MMSE	20	16	66.3	66.5	NR	NR	3	2	3	8
Okuda 1995 [10]	Japan	NR	52.4	H&Y, MMSE	42	44	67.4	70.3	8/14	11/11	2	1	3	6
Okuda 1996 [24]	Japan	NR	52.9	H&Y, MMSE	36	24	65.7	67.3	NR	NR	2	1	3	6
Peppe 1995 [27]	Italy	NR	13.3	UPDRS, H&Y	36	16	62.5	61.2	NR	NR	2	1	3	6
Quagliato 2014 [28]	Brazil	UKBB	83.76	UPDRS, H&Y	86	76	63.1	62.4	27/16	17/21	3	1	3	7
Sagliocco 1997 [17]	Austria	NR	31.25	H&Y, MMSE	34	33	61.94	54.53	8/9	9/8	2	1	3	6
Sartucci 2006 [3]	USA	UKBB	26.4	UPDRS, H&Y	21	24	60.1	46.8	6/6	6/6	3	1	3	7

PD = Parkinson's disease; HC = healthy controls; NR = not reported; UKBB = United Kingdom Brain Bank clinical diagnostic criteria; UPDRS = Unified Parkinson's Disease Rating Scale; H&Y = Hoehn and Yahr Scale; SE-ADL = Schwab and England Activities of Daily Living Scale; MMSE = Mini-Mental State Examination; SPES/SCOPA = Short Parkinson's Evaluation Scale/Scales for Outcomes in Parkinson's disease; CMA = Chinese Medical Association clinical diagnostic criteria; CNCESD = Chinese National Conference on Extra-Pyramidal System Disease diagnostic criteria; HDS = Hasegawa Dementia Scale.

**Table 2:** Metaregression analysis and subgroup analysis.

Subgroup factor	Assign criteria	Number of studies	WMD (95% CI)	*P*-difference between groups^*∗*^
Sample size	≤20	10	4.20 (1.38, 7.02)	0.001
	>20	10	7.64 (2.21, 13.1)	

Publication year	Before 2000	10	6.25 (1.91, 10.6)	0.12
	2000 or later	10	5.79 (0.49, 11.1)	

Age (years)	<64	9	9.39 (3.04, 15.7)	0.08
	≥64	11	3.38 (1.18, 5.58)	

Proportion of men	>50%	8	4.47 (2.03, 6.91)	0.03
	≤50%	4	8.44 (−3.98, 20.9)	

Duration (months)	≤56.4	8	4.82 (−2.31, 12.0)	0.01
	>56.4	7	6.38 (1.08, 11.6)	

^*∗*^Adjusted for all factors in this table.

## Supplementary Materials

Supplementary Materials PRISMA Checklist.
